# Paving the Way toward Successful Multiple Myeloma Treatment: Chimeric Antigen Receptor T-Cell Therapy

**DOI:** 10.3390/cells9040983

**Published:** 2020-04-16

**Authors:** Ewelina Grywalska, Barbara Sosnowska-Pasiarska, Jolanta Smok-Kalwat, Marcin Pasiarski, Paulina Niedźwiedzka-Rystwej, Jacek Roliński

**Affiliations:** 1 Department of Clinical Immunology and Immunotherapy, Medical University of Lublin, 20-093 Lublin, Poland; jacek_rolinski@wp.pl; 2 St. John’s Cancer Centre, 20-090 Lublin, Poland; 3 Department of Oncocardiology, Holy Cross Cancer Centre, 25-734 Kielce, Poland; spbasia@gmail.com; 4 Department of Clinical Oncology, Holy Cross Cancer Centre, 25-734 Kielce, Poland; jolantasmok1@gmail.com; 5 Department of Immunology, Faculty of Health Sciences, Jan Kochanowski University, 25-317 Kielce, Poland; marcinpasiarski@gmail.com; 6 Department of Hematology, Holy Cross Cancer Centre, 25-734 Kielce, Poland; 7 Institute of Biology, University of Szczecin, 71-412 Szczecin, Poland; paulina.niedzwiedzka@gmail.com

**Keywords:** multiple myeloma, chimeric antigen receptor T (CAR T), BCMA, immunotherapy

## Abstract

Despite the significant progress of modern anticancer therapies, multiple myeloma (MM) is still incurable for the majority of patients. Following almost three decades of development, chimeric antigen receptor (CAR) T-cell therapy now has the opportunity to revolutionize the treatment landscape and meet the unmet clinical need. However, there are still several major hurdles to overcome. Here we discuss the recent advances of CAR T-cell therapy for MM with an emphasis on future directions and possible risks. Currently, CAR T-cell therapy for MM is at the first stage of clinical studies, and most studies have focused on CAR T cells targeting B cell maturation antigen (BCMA), but other antigens such as cluster of differentiation 138 (CD138, syndecan-1) are also being evaluated. Although this therapy is associated with side effects, such as cytokine release syndrome and neurotoxicity, and relapses have been observed, the benefit–risk balance and huge potential drive the ongoing clinical progress. To fulfill the promise of recent clinical trial success and maximize the potential of CAR T, future efforts should focus on the reduction of side effects, novel targeted antigens, combinatorial uses of different types of CAR T, and development of CAR T cells targeting more than one antigen.

## 1. Introduction

Multiple myeloma (MM) is a cancer of plasma cells that build up in the bone marrow. MM results in hypercalcemia, anemia, renal dysfunction, bone destruction, and bone marrow failure. Even though MM has a relatively low prevalence (1% of all cancers and 10% of all hematological malignancies), it is the second most common hematological malignancy [1]. MM is usually diagnosed between the ages of 65 and 74 years, and the five-year survival rate is approximately 51% [2].

Current treatment options include glucocorticosteroids, standard chemotherapy (e.g., cyclophosphamide, doxorubicin), proteasome inhibitors (e.g., bortezomib, ixazomib), immunomodulatory drugs (e.g., thalidomide), histone deacetylase inhibitors (e.g., panobinostat), and monoclonal antibodies (e.g., duratumumab, elotuzumab) [3,4,5,6,7]. Novel treatment strategies such as proteasome inhibitors or monoclonal antibodies have led to remarkable improvements in doubling patient survival from four to eight years [8,9,10]. Unfortunately, despite the availability of therapeutic options, MM still has a very poor prognosis. One reason for this is that most patients with MM ultimately relapse and become unresponsive to currently available treatment options [11]. Such a population of patients (refractory individuals) is characterized by median survival (MS) of 13 months and median progression-free survival (PFS) of five months [12]. Therefore, durable and deep remission is the key objective of MM therapy [13].

Even when the availability of therapy is not a problem, the cost is not always affordable for patients with MM in every country [14]. Because MM therapy is mostly administered as a combination of three or more drugs and patients are continuously treated for years, the cost can range from $60,000 to $200,000 per year [15]. Therefore, there is a serious clinical need to develop more efficient and affordable treatment options.

One novel strategy to eliminate cancer is chimeric antigen receptor (CAR) T-cell therapy. CAR T cells are T cells from patients that are genetically re-engineered to present a CAR on their surface targeting tumor-specific antigens. As a result, CAR can bind to the desired antigen expressed on cancer cells and initiate cell lysis [16]. Thus, successful CAR development critically depends on selecting an optimal surface antigen present in cancer cells and absent in normal cells. So far, two CAR T-cell therapies have been approved by the US Food and Drug Administration (FDA) for the treatment of cancer patients: Axicabtagene ciloleucel (Yescarta^®^) and tisagenlecleucel (Kymriah^®^). Both of them target the cluster of differentiation 19 (CD19) antigen, and both treatments are approved for subsets of patients with relapsed or refractory large B-cell lymphoma. Additionally, Kymriah^®^ is also approved for children and young adults with acute lymphoblastic leukemia. The reported response rates are 68–93% in acute lymphoblastic leukemia (ALL), 57–71% in chronic lymphocytic leukemia, and 64–86% in B-cell lymphoma [17]. The remarkable achievements of CAR T-cell therapy in the treatment of relapsed and refractory ALL and chronic lymphocytic leukemia have encouraged the development of CAR T cells for the treatment of MM [18,19,20,21].

Currently, multiple antigen targets are being studied in clinical trials with MM patients. The results of some of these trials have been published, as in the case of B-cell maturation antigen (BCMA), cluster of differentiation (CD) 19 (CD19), CD138, Natural killer group 2 member D (NKG2D), and kappa light chain antigens. Many trials are ongoing, as in the case of CD38, signaling lymphocytic activation molecule (SLAM) family member 7 (SLAMF7), CD44 variant 6 (CD44v6), CD56, G-protein-coupled receptor class C group 5 member D (GPRC5D), transmembrane activator and calcium-modulator and cyclophilin ligand (CAML) interactor (TACI), and New York esophageal squamous cell carcinoma 1 (NY-ESO-1). Some antigens, such as CD229 and integrin β7, are in the preclinical stage. Unfortunately, so far there is no FDA-approved CAR T-cell therapy for MM. Currently, the use of CAR T cells in the treatment of MM is limited to selected antigens in phase I clinical studies, but in the future this treatment strategy could fill the gap in the treatment landscape. Here we discuss the recent advances of CAR T-cell therapy for MM with a focus on currently evaluated targeted antigens, future development directions, and possible adverse effects.

## 2. Structure and Mechanism of Action of CAR T Cells

**Figure 1:**
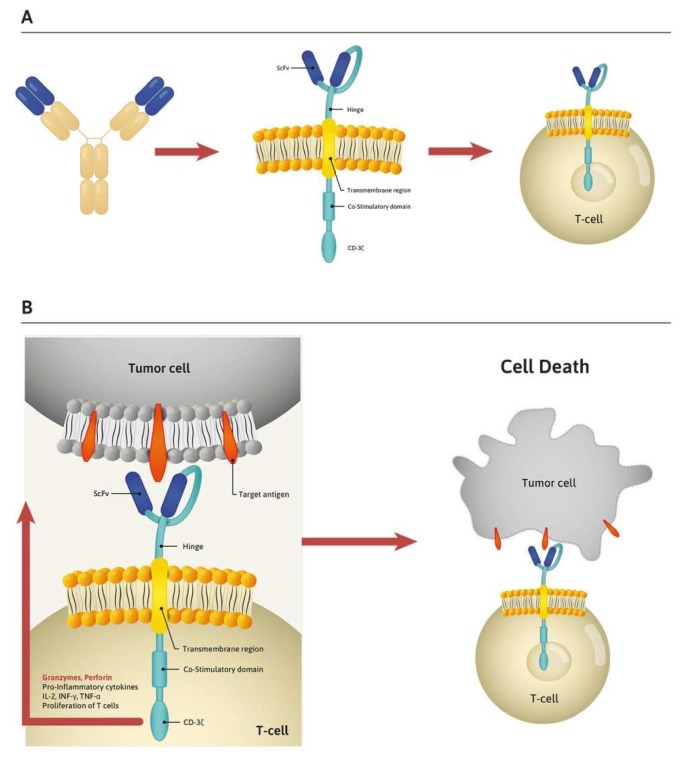
(**A**) The most important part of a chimeric antigen receptor (CAR) is a single-chain variable fragment (scFv), which originates from variable light and heavy chains of monoclonal antibody. This antibody-like surface fragment is tumor antigen specific, and together with signaling regions from the T-cell receptor is expressed on the surface of T cells forming CAR. (**B**) CAR binds its targeted tumor antigens, which induces the release of cytotoxic granules and cytokines leading to lysis of tumor cells.

CAR is composed of three parts: an extracellular antibody-like surface domain, a transmembrane domain, and an intracellular signaling domain [22]. The antibody-like surface domain consists of heavy and light chains originating from an antibody to generate a single-chain variable fragment (scFv) [23]. The development of scFv by Eshhar et al. which resulted in T-cell activation, is considered as a starting point of the development of CAR T cells [24]. The transmembrane domain originates from immunoglobulin G4 (IgG4) or CD8, while the intracellular part consists of a costimulatory domain and the CD3 zeta cytoplasmic domain (CD3ζ) and is responsible for T-cell activation. The first-generation CAR had only the CD3ζ domain [25,26]. To enhance survival, improve activation, and expand the modified T cells, the CD3ζ domain was coupled with additional signaling domains, such as CD28 or 4-1BB cytoplasmic domain (CD137), an activation-induced costimulatory molecule, forming second-generation CARs [27,28]. Such modified CARs laid the foundation for currently available CAR T-cell therapy (Figure 1A). The working principle of CAR T cells is based on the binding reaction between the CAR and its targeted antigens, which induces the release of cytokines and cytotoxic granules, leading to the destruction of cancer cells (Figure 1B). The intracellular part of third-generation CAR T cells takes advantage of the signaling capabilities of two costimulatory domains (e.g., CD28 and 4-1BB). The fourth-generation CAR T cells, T cells redirected for universal cytokine-mediated killing (TRUCKs), have been generated by further modifications, including, for example, transgenes for the secretion of cytokines such as interleukin-12 (IL-12) [29,30].

## 3. Clinical Trials

### 3.1. BCMA

B-cell maturation antigen (BCMA) is generally present on the surface of mature B lymphocytes, with minimal expression in hematopoietic stem cells and nonhematopoietic tissue [31]. BCMA is overexpressed in MM cells; however, its expression level can be different among clinical samples of patients [32]. BCMA is involved in survival, proliferation, and drug resistance of MM cells [33,34] and, therefore, can be used as a marker for the disease course and disease outcomes [35]. As well as the surface-expressed form, BCMA also has a soluble variant present in the peripheral blood of patients with MM [36]. It was shown that the administration of soluble BCMA lowered immune responses [37,38]. As a result, soluble BCMA may theoretically negatively influence BCMA-targeting CAR T cells [39].

Based on preclinical studies in which BCMA-targeted CAR T cells exhibited strong efficacy, those cells initialized worldwide CAR T cell clinical studies. To date, BCMA CARs have allowed the most valuable clinical data to be obtained in MM (Table 1). Gagelmann et al. performed a meta-analysis of 15 studies comprising a total of 285 patients with heavily pretreated relapsed/refractory MM (RRMM) [40]. This analysis showed a pooled overall response of 82% and a complete response of 36%. Severe cytokine release syndrome (CRS) grade 3–4 and neurotoxicity occurred in 15% and 18% of cases, respectively.

The first-in-human phase I clinical study of anti-BCMA CAR T cells was conducted in patients with RRMM (NCT02215967) [41]. A total of 24 patients were included in this study, and the following doses were examined: 0.3 × 10^6^, 1 × 10^6^, 3 × 10^6^, and 9 × 10^6^ CAR T cells/kg. This study showed minimal antitumor activity of the lowest doses of (0.3–3.0) × 10^6^ CAR T cells/kg with an overall response rate (ORR) of 20%. For 16 patients treated with the highest dose of 9 × 10^6^ CAR T cells/kg, the ORR was 81%, but cytokine release syndrome (CRS)-related toxicities were observed. Moreover, it was shown that soluble BCMA did not negatively affect the efficacy of anti-BCMA CAR T cells. As a result, this study proved that BCMA is a unique target for plasma cell malignancies and demonstrated the feasibility of anti-BCMA CAR T-cell therapy, providing directions for future development.

#### 3.1.1. The bb2121

The bb2121 is a second-generation BCMA-targeted CAR T-cell construct containing anti-BCMA scFv and 4-1BB and CD3ζ domains [42]. In a phase I study (CRB-401, NCT02658929), bb2121 was injected into 33 patients with RRMM [43]. Safety was the primary endpoint. All patients received chemo-conditioning with cyclophosphamide and fludarabine before injection with a single dose of CAR T cells. The study design included a dose-escalation phase, in which doses of 50 × 10^6^, 150 × 10^6^, 450 × 10^6^, or 800 × 10^6^ CAR T cells were infused in patients, and a dose-expansion phase, in which doses of 150 × 10^6^ to 450 × 10^6^ CAR T cells were infused. Cytopenia was the most frequently observed severe adverse event (SAE); 25 out of 33 patients (76%) had CRS grades 1–2 and two had grade 3. CAR T-cell-related encephalopathy syndrome (CRES) was observed in 42% of patients. The ORR was 85%. Nine percent of patients achieved a complete response (CR) and 36% achieved stringent complete response (sCR). Six of the CR patients relapsed. The progression-free survival (PFS) was 11.8 months. The bb2121 CAR T cells were detectable up to one year after infusion. Additionally, a correlation between CAR T-cell expansion and clinical outcomes was observed. Further reliable data on the efficacy of bb2121 should be provided by an ongoing phase III study (NCT 03651128).

#### 3.1.2. The bb21217

The bb21217 is the updated version of bb2121. Both have a similar structure, but bb21217 contains an extra domain encoding a phosphatidylinositol 3-kinase (PI3K) inhibitor. This modification led to an improvement of CAR T cells in terms of persistence and potency. A phase I first-in-human multicenter dose-escalation trial of bb21217 CAR T cells in patients with RRMM (CRB-402 (NCT03274219)) recruited 12 patients with high BCMA expression levels on MM cells [44]. The CAR T cell doses were similar to those in the CRB-401 study (NCT02658929). This study aimed to assess the safety, efficacy, and duration of the bb21217 effect. The results showed one sCR, three very good partial responses (VGPRs), and two partial responses (PRs). The ORR was 83%. The CAR T cells were detectable six months after administration.

#### 3.1.3. LCAR-B38M

LCAR-B38M is a bispecific CAR T-cell product containing a CAR with scFv recognizing two BCMA epitopes: clones VHH1 and VHH2 [45]. LCAR-B38M has been studied in patients with advanced RRMM in the LEGEND-2 trial (NCT03090659), a single-arm open-label multicenter study [46]. LCAR-B38M CAR T cells were infused three times (20%, 30%, and 50% of the total dose; median CAR T-cell dose was 0.5 × 10^6^ cells/kg) into 57 patients at the Second Affiliated Hospital of Xi’an Jiao Tong University. The ORR was 88%. CR was achieved by 39 patients (68%, all were minimal residual disease (MRD)-negative), VGPR was achieved by three patients (5%), and PR by eight patients (14%). Additionally, the median time to response was one month. LCAR-B38M CAR T cells were undetectable in the peripheral blood of 71% of patients with MM at month 4. Also, 10 months after the infusion, CAR T cells were confirmed in only five patients. Adverse events (AEs) with the highest prevalence were as follows: Pyrexia (91%), CRS (90%), thrombocytopenia (49%), and leukopenia (47%). The most frequently observed CRS grades were 1 and 2 (83%); grade 3 was reported in four patients with MM (7%). At the other three sites in China, 17 patients were infused with LCAR-B38M CAR T cells ((0.21–1.52) × 10^6^ cells/kg) (ChiCTR-ONH-17012285) [47]. In this study, three infusions (n = 8) were compared with one infusion (n = 9) of the total CAR T dose. Among compared groups, no differences in patient responses were reported and similar toxicities were found. The ORR was 88.2% and PFS rates were 82.4% and 52.9% at 6 and 12 months, respectively, with a one-year overall survival (OS) rate of 82.3%. Varying severity of CRS was observed in 16 patients. One case of patient death as a result of tumor lysis syndrome and CRS was reported. No correlation was observed between disease relapse and characteristics such as gender, age, cytogenetic markers, conditioning scheme, CAR T-cell infusion dosage, or delivery method. The results of this trial suggest that LCAR-B38M has the potential to become an efficient immunotherapeutic agent for RRMM.

#### 3.1.4. P-BCMA-101

P-BCMA-101 is a CAR T-cell construct created by fusing an anti-BCMA Centyrin™ with a CD3ζ/4-1BB domain, which results in CARTyrin. Centyrins, a class of alternative scaffold proteins based on a consensus fibronectin domain, are currently used to design novel biological therapeutics. CARTyrin is a non-immunoglobulin-based scaffold centyrin molecule generated with the use of a non-viral piggyBac transposon-based delivery system [48]. A major advantage of not using a viral vector system is lower costs. Moreover, centyrins are fully humanized and have strong binding affinities, and as a result are more stable and less immunogenic. The safety and efficacy of P-BCMA-101 have been tested in a phase I clinical trial with a single administration and dose escalation (NCT03288493) [49]. Nineteen extensively pretreated patients with RRMM, assessed as high risk, were treated with (48–430) × 10^6^ P-BCMA-101 CAR T cells. Nine patients with MM completed their first two-week evaluation; one patient had sCR, one presented non-secretory disease near the CR of plasmacytomas by positron emission tomography/computed tomography (PET/CT), one had VGPR, and five had PR. The ORR was 43%. Grade 2 CRS occurred in only one patient. No deaths and no CRES were observed, indicating that the treatment was well tolerated.

#### 3.1.5. JCARH125

JCARH125 is composed of a lentiviral CAR construct with a fully humanized scFv, optimized spacer, and 4-1BB and CD3ζ domains [50]. JCARH125 was evaluated in the EVOLVE (NCT03430011) trial, a multicenter phase I/II clinical trial, in patients with RRMM [51]. In this study, JCARH125 was administered in a single dose to patients in each cohort on day 1. Dose escalation was decided with the use of modified toxicity probability interval 2 (mTPI-2). The first two dose levels assessed were 50 and 150 × 10^6^ CAR T cells. Each dose level will be assessed on a minimum of three patients. Forty-four patients with MM were enrolled. The ORR was 82%, and 35 patients had CRS. The study is limited by its small patient population and short follow-up; hence, longer follow-up and more patients are necessary. The study is ongoing.

#### 3.1.6. CT053

CT053 are genetically engineered T cells with an extracellular anti-BCMA human scFv and 4-1BB costimulatory domain. CT053 CAR T cells were investigated in a multicenter clinical study (NCT03915184) [52]. In all patients, BCMA expression was confirmed in MM cells. A single dose of CAR T cells was planned. The second dose will be infused if clinically needed. In total, 16 patients were infused with CT053 cells, the majority with a single dose of 1.5 × 10^8^ cells. Thirteen of the 16 patients achieved the assessment point and were categorized as having CR (three patients), VGPR (six patients), and PR (four patients). CRS was reported in three patients. No CRES was observed. Median follow-up was 8 weeks (4 to 36 weeks), and in this period an ORR of 100% was obtained for 13 analyzed patients. In 11 of those 13 patients, CT053 CAR T cells were present for up to 4–6 months. These results suggest that CT053 cells are an interesting subject for further investigation in RRMM therapy.

#### 3.1.7. MCARH171

MCARH171 is composed of a humanized scFv, a 4-1BB costimulatory domain, and a truncated epidermal growth factor receptor safety system. MCARH171 was studied in a phase I dose-escalation trial to evaluate its safety and efficacy in patients with RRMM (NCT03070327) [53]. In total, 11 patients were treated with MCARH171 cells under a 3 + 3 dose design. Patients received one of the following doses per cohort: 72 × 10^6^, 137 × 10^6^, 475 × 10^6^, or 818 × 10^6^ CAR T cells. The ORR was 64%. The median response duration was 106 days. In four (40%) and two (20%) patients, CRS grades of 1–2 and 3 were reported, respectively. The patients who were infused with lower doses of MCARH171 cells (72 × 10^6^, 137 × 10^6^) had lower peak peripheral blood expansion compared to those infused with higher doses (475 × 10^6^, 818 × 10^6^). Moreover, 16.7% of patients (1 of 6) infused with lower doses and 60% of patients (3 of 5) infused with higher doses had a clinical response lasting less than 6 months. This study shows that MCARH171 has an acceptable safety profile.

#### 3.1.8. BRD015

BRD015 is composed of a lentiviral CAR with a murine anti-BCMA scFv and CD28 co-stimulation domain. BRD015 has been studied in a phase I trial (ChiCTR-OPC-16009113) conducted in Tongji Hospital of Tongji Medical College, China. This study involved 28 patients with MM. Doses of (5.4–25.0) × 10^6^ CAR T cells/kg were infused in patients. Twenty-two patients with MM were divided into two groups based on the BCMA expression level on MM cells. For the high-BCMA group (73% CR) the ORR was 87%, and for the low-BCMA group (33% CR or VGPR) the ORR was 100%. Among 28 evaluable patients, 26 achieved remission. Moreover, a positive correlation between clinical responses and peak blood CAR T-cell levels was observed [54].

#### 3.1.9. CT103A

As mentioned above, BRD015 has the murine BCMA epitope, which causes problems with CAR T-cell therapy. To solve this problem, a novel anti-BCMA CART, CT103A, was developed. CT103A is composed of a lentiviral vector with a fully human BCMA scFv and a 4-1BB domain. CT103A was studied in a single-center phase I study with a 3 + 3 dose-escalation design (ChiCTR1800018137). In this study, (1–6) × 10^6^ cells/kg of CAR T cells were administered to nine patients with RRMM; three of the patients had undergone previous BRD015 therapy but had relapsed [55]. CT103A cells were infused in three doses: 1 × 10^6^, 3 × 10^6^, and 6 × 10^6^ cells/kg. The ORR was 100%, and a response was observed within 14 days. After the first two dose levels, mild CRS was observed. Interestingly, after CT103A therapy, two of the three patients who relapsed after BRD015 therapy achieved CR and one patient achieved VGPR.

### 3.2. CD19

CD19 is a member of the immunoglobulin superfamily expressed on the surface of many B cell malignant cells in the course of, e.g., acute and chronic lymphocytic leukemia [56]. It is worth mentioning that CD19 is not an ideal antigen for the treatment of MM because it is rarely present on MM cells. In recently conducted studies, CD19 was discovered on a minor MM stem cell subset [57]. The CD19-engineered CAR T-cell product tisagenlecleucel has been approved for the treatment of advanced B-cell malignancies. It was shown that tisagenlecleucel infusion resulted in a long-lasting full response in one RRMM patient after being treated with a high dose of melphalan and autologous stem cell transplantation (ASCT) [58]. The response was surprising because of the lack of CD19 on 99.95% of myeloma cells in the patient. Moreover, the response lasted even after tisagenlecleucel was no longer present in the blood. This indicated that the continuous presence of CAR T cells in the blood is not required for a sustainable response. This therapeutic strategy was also examined in another clinical study (NCT02135406). Complete data of this trial, including 10 patients with MM treated with tisagenlecleucel following administration of high-dose melphalan and ASCT, have been published [59]. Enrolled patients had previously undergone ASCT but experienced disease progression within one year. This study showed that treatment with ASCT + tisagenlecleucel was safe. No severe CRS was reported. The observed toxicity was mostly due to ASCT. Two patients had better PFS after ASCT + tisagenlecleucel (CTL019) compared with ASCT alone.

### 3.3. Combination of BCMA- and CD19-Targeted CAR T

The strategy to combine CAR T cells with different targets in one infusion has been used to solve the problem of antigen loss and inefficiency of CAR T therapy [60,61]. The safety and efficacy of combined infusion of BCMA- and CD19-targeted CAR T cells were evaluated in patients with RRMM (NCT 03196414) [62]. The third-generation CAR used in that study was composed of anti-BCMA and anti-CD19 scFv, OX40 (CD134), and CD28 costimulatory domain, and a CD3ζ signaling domain. First, eight patients with RRMM were infused with 1 × 10^7^/kg anti-CD19 CAR T cells (day 0). Then, split-dose infusions were performed: On day 1, each patient was infused with 40% anti-BCMA CAR T cells, and the rest of the cells (60%) were given on day 2. All eight patients had CRS. Because of many compounding factors in the study (such as haploidentical T cells), the outcome was difficult to assess. The same group used the strategy of combined CAR T therapies and administered anti-BCMA and anti-CD19 CAR T cells to patients with RRMM from day 14 to day 20 after ASCT (SZ-MM-CART02 study, NCT03455972) [63]. CAR T cells played a role in post-transplant consolidation therapy. The dosage and administration were equal to those used in the first trial. Nine patients were enrolled. The median follow-up was three months. In all patients, BCMA was present on MM cells, but no CD19 presence was confirmed. The ORR was 100%. Three CRs, two VGPRs, and four PRs were reported. After CAR T infusion, the treatment response improved to three CRs and six VGPRs. Minimal residual disease (MRD) changed from 37.5 to 66.7%. Mild grade 1–2 CRS was observed in all patients. A more than 1000-fold expansion at peak level was reported and a 100-fold increase in a similar cohort. It has been suggested that anti-CD19 CAR may improve T-cell expansion in vivo at the expense of B-cell hypoplasia. Therefore, the anti-CD19 CAR T cells present in the combination may result in a substantial expansion of anti-BCMA CAR T cells. Because the approach of combined administration of CAR T cells may be responsible for higher toxicity related to the parallel targeting of many antigens, it can also improve T-cell activation and enhance tumor lysis. However, it is still difficult to draw any conclusions about these early combination CAR T-cell therapy approaches.

### 3.4. NKG2D

Natural killer group 2 member D (NKG2D) is a cell surface protein present on NK cells, CD8+ T cells, NK/T cells, and γδ T cells [64]. Of note, under normal conditions, NKG2D ligands are not present on the surface of nonmalignant cells. However, neoplastic transformation induces the expression of NKG2D ligands [65]. A phase I first-in-human clinical study with CAR T cells recognizing multiple NKG2D ligands was performed by Baumeister et al. [66]. The study included five patients with RRMM and seven patients with acute myeloid leukemia/myelodysplastic syndrome. CRS and neurotoxicity were not observed. The persistence of NKG2D CAR T cells was low, and no clinical effects were detected. The reasons for the treatment failure are unclear. One possible cause is that no lymphodepleting chemotherapy was given to patients before CAR T-cell administration; it was previously shown that lymphodepletion is crucial for CAR T-cell engraftment and clinical efficacy [67]. Another reason for the lack of response could be that a first-generation CAR was used. Another possible reason for the lack of clinical activity is related to the cell type used for this treatment. It was demonstrated that only anti-NKG2D CAR NK cells are capable of eliminating MM cells [68].

### 3.5. Kappa Light Chain

Attenuated humoral immunity is a common consequence of anti-CD19 CAR T-cell therapy. Thus, a more specific CAR T product that would save some B cells and consequently preserve humoral immunity is needed. Due to the expression of either κ or λ light chains by mature B cells, one of these two B cell populations can be set as a target, leaving the other population alone. In consequence, this approach could be employed to lyse only selected types of MM cells expressing one type of light chain; in this case, B cells expressing the second type of light chain would not be lysed. Possible limitations are as follows: Immunoglobulin secretion by plasma cells into the bloodstream instead of presentation on the surface, and B cell depletion in the majority of patients awaiting CAR T-cell therapy associated with previous treatments. This makes studies of therapy based on selective light chain very challenging.

Ramos et al. designed a kappa-specific CAR that was capable of recognizing kappa-restricted MM cells [69]. Because cases of MM cells with surface immunoglobulins have been confirmed [70], the kappa light chain can be a promising target for MM. A phase I trial (NCT00881920) included seven patients with MM and nine patients with non-Hodgkin lymphoma. [69]. No objective responses were observed in the seven MM patients: MRD remained stable for 17 months in one patient, another patient had stable disease for two years, and two others had transient stable disease. In one patient, after 1.5 years (following conventional therapy), CAR T-cell treatment was repeated and again produced the transient stable disease. The other three patients did not respond to CAR T-cell therapy. Additionally, no severe CRS was confirmed, and no other side effects were reported. Because light chains are mostly secreted, this may limit CAR T-cell targeting of cancer cells and, as a result, cause CAR T-cell reduction.

### 3.6. CD38

CD38 is a transmembrane glycoprotein first detected on B lymphocytes in mice [71]. Generally, CD38 is present on precursors of B cells, plasma cells, NK cells, T cells, and myeloid precursors. CD38 is also expressed on muscle cells, prostate cells, gut, and osteoclasts [72]. Because CD38 is abundantly present in MM cells [73], several anti-CD38 monoclonal antibodies have been examined in patients with MM. The first FDA-approved human anti-CD38 monoclonal antibody for RRMM treatment is daratumumab. Daratumumab’s anti-MM activity is based on antibody-dependent T-cell-mediated cytotoxicity, antibody-dependent phagocytosis, and complement-dependent cytotoxicity [74]. Isatuximab (SAR650984) is another anti-CD38 monoclonal antibody with preclinical and clinical activity against MM cells [75]. Data from daratumumab and isatuximab support the feasibility of developing anti-CD38 CAR T cells. These results indicate that anti-CD38 CAR T cells could proliferate, produce cytokines, and destroy CD38+ MM cells. However, the limitation is that these anti-CD38 CAR T cells lack specificity in terms of lysis; they lyse not only MM cells, but also normal cells expressing CD38. Light-chain exchange technology has been proposed to overcome this problem [76]. The first phase I clinical trial testing anti-CD38 CAR T cells was carried out in 2018 at the University of Pennsylvania and the Roger Williams Medical Center (NCT03464916). Apart from studying CD38-targeted CAR T cells as monotherapy for patients with RRMM (NCT03464916), these cells were also investigated in combination with BCMA (NCT03767751), with CD19 (NCT03125577), with BCMA, CD138, or CD56 (NCT03473496, NCT03271632), and with BCMA and NY-ESO-1 (NCT03638206).

### 3.7. NY-ESO-1

New York esophageal squamous cell carcinoma 1 (NY-ESO-1) antigen is a member of the family of cancer/testis (CT) antigens found in many types of cancers. NY-ESO-1 has been found in up to 60% of patients with relapsed MM. This antigen is an intracellular oncoprotein that can be used as a target for T-cell receptor (TCR)-engineered T-cell therapy in MM [77]. NY-ESO-1 has the potential to overcome the major limitation of CAR T-cell therapy, applicability to only cell surface antigens. Intracellular antigens are usually found in the context of human leukocyte antigen (HLA) molecules and are identified by TCRs.

CD8+ effector CAR T cells, which recognized the HLA-A*02- 01-NY-ESO-1157-165 peptide complex, were constructed [78]. In this study, anti-NY-ESO-1 CAR T cells recognized NY-ESO-1 present on MM cells. What is more, some of these T cells secreted interferon gamma (IFNγ) upon stimulation by NY-ESO-1. As a result, NY-ESO-1 can be considered as a promising target for MM therapy. In a phase I/II trial, anti-NY-ESO-1 CAR T cells were studied in 20 patients with MM. This study showed good clinical responses in 16 of the 20 patients (80%) at an advanced stage of the disease. Median PFS was 19.1 months [77].

### 3.8. SLAMF7

SLAMF7 is a signaling lymphocytic activation molecule (SLAM) family member, first found on the surface of NK cells [79]. Apart from NK cells, SLAMF7 is present on a fraction of T cells, B cells, dendritic cells, and macrophages. Moreover, it has been observed on 95% of normal and malignant plasma cells [80]. Because SLAMF7 is absent in non-hematologic organs and hematopoietic stem cells, it is a potential CAR target in MM therapy. Indeed, SLAMF7 is actively studied as a target for MM therapy [81,82]. The introduction of elotuzumab, a humanized immunoglobulin G kappa (IgG-κ) antibody targeting SLAMF7, accelerated the studies of SLAMF7 as a target antigen for CAR T cell therapy [83]. In 2015 the FDA approved elotuzumab combined with lenalidomide and dexamethasone for the treatment of patients with MM.

Anti-SLAMF7 CAR T cells were designed by Gogishvili et al., who obtained scFvs from elotuzumab [84]. After 20 h of co-culture with myeloma cells, anti-SLAMF7 CAR T cells exerted antitumor activity, resulting in lysis of more than 90% of myeloma cells. Additionally, in the case of CD38+ CD138+ SLAMF7+ malignant cells from patients with MM, anti-SLAMF7 CAR T cells showed destruction of these cells in four hours. The limitations of anti-SLAMF7 CAR are associated with the presence of SLAMF7 in a fraction of normal cells. It was shown that anti-SLAMF7 CARs can recognize SLAMF7 highly expressed on T cells. It has also been shown that T cells with lower SLAMF7 expression can be preserved, and these cells can induce an immune response while remaining functional [84].

To date, three CAR T-cell constructs targeting SLAMF7 have been studied in several clinical trials. One study assessed T cells lentivirally transduced to express an anti-SLAMF7 CAR molecule (NCT03710421). What is interesting is a safety system of this CAR construct based on the truncated epidermal growth factor receptor (EGFRt) molecule. This system can be used for CAR T-cell depletion in the case of severe side effects and is based on the cetuximab/anti-EGFR monoclonal antibody [85]. Another study is a phase I/II clinical trial, part of the CARAMBA project of the European Union Horizon 2020 program (https://www.caramba-cart.eu/). The CAR T cells used in that study contain the same safety system, but the unique feature of this construct is its nonviral, Sleeping Beauty transposon-based method to introduce the CAR gene into T cells. Another study is evaluating healthy, allogeneic anti-SLAMF7 CAR T cells (UCARTCS1) [86].

### 3.9. CD44 Variant 6

CD44 is a glycoprotein present on epithelial and hematologic cancer cells. CD44 isoform variant 6 (CD44v6) has been detected in about 43% of MM cases [87]. In a phase I radioimmunotherapy trial, it was shown that a humanized monoclonal antibody targeting CD44v6, bivatuzumab, has an acceptable safety profile [88]. It has been observed that the main side effect of bivatuzumab is skin toxicity. Unfortunately, because one fatal drug-related adverse event (AE) occurred before reaching the maximum tolerated dose, further development was terminated. Casucci et al. constructed and tested anti-CD44v6 CAR T cells that do not recognize hematopoietic stem cells and keratinocytes but can cause reversible monocytopenia [89]. A multicenter phase I/II first-in-human clinical trial of anti-CD44v6 CAR T cells in patients with MM is ongoing European Endeavour for Chimeric Antigen Receptor Therapies (EURECART).

### 3.10. CD56

CD56 is a glycoprotein and a member of the immunoglobulin superfamily [90]. A high expression level of CD56 on malignant plasma cells has been observed in up to 78% of patients with MM. Additionally, CD56 is present on the surface of NK cells, neural cells, and epithelial cells, and in normal tissues in subpopulations of activated T cells. Lorvotuzumab (HuN901) is a humanized monoclonal antibody that targets CD56. This antibody demonstrated high anti-myeloma cell activity both in vitro and in vivo [91]. Lorvotuzumab mertansine (LM) is an antibody-drug conjugate targeting CD56+MM cells. Elevated antitumor activity against CD56+MM cells has been shown in the case of single-agent LM or LM administered with lenalidomide and dexamethasone [92]. Benjamin et al. studied CAR T cells that specifically targeted MM cells in a preclinical study [93]. However, the limitation of anti-CD56 CAR T cells is their possible neurologic toxicity because of the presence of CD56 in cells of the central and peripheral nervous system.

### 3.11. CD70

CD70 (CD27L) belongs to the tumor necrosis factor family. CD70 is present on some solid and hematologic malignancies, including MM [94]. Due to low CD70 expression on normal cells, this can be a promising target for anticancer therapy. SGN-70 is a humanized anti-CD70 antibody mediating antitumor activity in vivo [95]. Apart from SGN-70, another specific monoclonal antibody against CD70 is BMS-936561 (MDX-1203) [96]. Results from a first-in-humans multicenter phase I study of BMS-936561 showed an acceptable safety profile. Also, observations from a preclinical study additionally supported the efficacy and safety of a CAR targeted to CD70-expressing tumors [97]. It has been reported that anti-CD70 CAR T therapy led to strong antitumor responses in human cancer cells and animal models [98], but the antitumor effect in MM is not yet fully understood.

### 3.12. CD138

CD138 (syndecan 1) is a membrane protein that belongs to the syndecan family of heparan sulfate proteoglycans [99]. While CD138 is present on most malignant and normal plasma cells, it is not detectable on other hematopoietic cells such as T and B cells [95]. Because CD138 plays a key role in cell proliferation, infiltration, and apoptosis of MM cells, it is a promising candidate target for CAR T cell MM treatment [100]. CD138 antibody (BT062, indatuximab) was used in the treatment of patients with MM. In a phase I/II clinical trial of BT062, a clinical response was reported in only 1 out of 23 patients [101]. The combination of BT062 with lenalidomide increased the overall response rate by up to 83% [102]. A first-in-human trial of anti-CD138 CAR T-cell therapy was performed in the Chinese People’s Liberation Army (PLA) General Hospital (NCT01886976) [103,104]. Five patients with RRMM previously treated with chemotherapy and stem cell transplantation were enrolled in this study. All patients received a CAR T-cell infusion with an average dose of 0.7563 × 10^7^ cells/kg. The results of this study showed that four of five patients had stable disease for more than three months, and one patient had a reduction of MM cells in the peripheral blood from 10.5% to less than 3% [104]. Only two grades of cytokine release syndrome (CRS) were observed. Due to the small sample size, it is hard to draw a solid conclusion, but an acceptable safety profile and limited antitumor activity provide a basis for further studies.

In 2019 Sun et al. provided data that strongly support the aforementioned clinical trials [105]. They used retroviral vector-mediated transduction of anti-CD138 scFvs from the CD138 antibody (BT062) to develop anti-CD138 CAR T cells. Then these cells were co-cultured with MM cells for three to five days in vitro. The result of this experiment was the destruction of CD138+ cells. Another experiment was based on co-culture of anti-CD138 CAR T cells with myeloma cells from patients with MM. Anti-CD138 CAR T cells from patients and healthy individuals showed antitumor activity against CD138+ malignant cells and, interestingly, against CD138+ stem cells. Additionally, it was shown that the antitumor effect was unchanged even in the presence of a high level of soluble CD138, which can inhibit CAR T cells by blocking the scFv domain. Of note, no lysis of epithelial and endothelial cells occurred. Also, no AEs were observed. Another clinical trial of anti-CD138 CAR T-cell therapy in patients with RRMM is ongoing (NCT03672318).

### 3.13. GPRC5D

Orphan G-protein-coupled receptor, class C group 5 member D (GPRC5D) is now a potential alternative option for MM treatment due to its upregulated mRNA level associated with poor MM prognosis [106]. It was shown that the expression of GPRC5D in patients with MM is restricted to three anatomical locations: Hair follicles, lung tissue, and bone marrow [107]. Although the presence of GPRC5D mRNA was confirmed [106], no protein product was detected in plasma cells of patients with MM [108]. What has been confirmed by gene chip profiling is that GPRC5D expression on malignant and normal plasma cells in bone marrow is 500-fold higher than that on plasma cells in the peripheral blood.

As discussed at the beginning of this paper, antigen-specific scFv is essential for the development of efficient CAR T cells. Anti-GPRC5D CARs were manufactured via retroviral vector-mediated transduction after selecting scFvs with high GPRC5D-specific affinity from a human B-cell-derived phage display [108]. Preliminary results show that the antitumor activity of anti-GPRC5D CAR T cells is comparable to that of anti-BCMA CAR T cells both in vitro and in vivo. Moreover, these cells had similar secretion profiles of cytokines such as interferon gamma (IFN-γ), tumor necrosis factor alpha (TNF-α), and granulocyte-macrophage colony-stimulating factor (GM-CSF). Importantly, even in relapsed xenograft models in cases of BCMA loss after anti-BCMA CAR T-cell therapy, anti-GPRC5D CAR T cells exhibited antitumor activity and, as a result, limited disease progression [108]. Because there is no relationship between the expression of GPRC5D and BCMA, anti-GPRC5D CART cells can potentially help patients experiencing a BCMA-loss relapse after anti-BCMA CAR T-cell therapy. These preliminary results on GPRC5D as a potential target in MM treatment led to a phase I clinical trial (MCARH109) to investigate anti-GPRC5D CAR T-cell therapy in patients with RRMM, including those who have undergone anti-BCMA therapy [109].

### 3.14. TACI

Transmembrane activator and calcium-modulator and cyclophilin ligand (CAML) interactor (TACI), a member of the tumor necrosis factor receptor superfamily, is present on malignant plasma cells, but usually less frequently than BCMA [110]. A proliferation-inducing ligand (APRIL) is a naturally occurring ligand for both BCMA and TACI [111]. APRIL-based CAR T cells, targeting BCMA and TACI on MM, have been developed [112]. Preclinical studies performed by Lee et al. showed that APRIL-based CAR T cells can destroy MM cells expressing BCMA and TACI as well as only TACI. These results suggest that APRIL-based CAR T-cell therapy can be especially valuable in the case of BCMA downregulation. BCMA downregulation is a tumor escape mechanism frequently reported in BCMA-targeted CAR T-cell studies [110]. Moreover, because TACI is also present on regulatory T (Treg) cells in patients with MM, APRIL-based CAR T cells can target MM cells directly, as well as indirectly by suppressing Treg cells [113]. Clinical studies are ongoing (NCT03287804).

### 3.15. SLAMF3

SLAMF3 is a receptor belonging to the SLAM family whose expression level on MM cells is unchanged regardless of the disease stage and treatment used. SLAMF3 is crucial for the survival of MM cells [114]. The first anti-SLAMF3 CAR T cell product was developed by Atanackovic et al. [114]. This CAR T cell construct showed high cytotoxic activity against SLAMF3-positive myeloma cells, and weak activity against B cells and resting T cells. A mouse model engrafted with luciferase-expressing U266MM cells provided interesting data about anti-SLAMF3 CAR T cell performance. While mice treated with anti-CD19 CAR T cells showed a strong bioluminescence signal after 18 days, no signal was detected for mice treated with anti-SLAMF3 CAR T cells, meaning that all U266MM cells were destroyed [114,115].

## 4. Preclinical Studies: Integrin β7, and CD1d as Future Targets

Because of the difficulties in identifying a targeted antigen specific only to myeloma cells, scientists have focused their attention on non-cancer-specific antigens that become cancer-specific after post-translational modification, such as glycosylation. As a result, integrin β7 was chosen as a promising target for MM [112]. MMG49 is a monoclonal antibody capable of specifically binding to integrin β7 in a cancer-specific conformation. MMG49 targets the activated state of the configuration-sensitive epitope of integrin β7, which is abundantly present on MM cells. In vitro studies have shown that MMG49 containing CAR T cells was able to proliferate, secrete interferon γ and IL-2, and damage MM cells. No MM cells escaped the therapy. Additionally, normal hematopoietic cells were unaffected. Clinical trials will start after the mouse-derived scFv is humanized.

CD1d is a major histocompatibility complex (MHC) class I-like molecule frequently present on premalignant and early MM cells; however, the expression level decreases along with the progression of the disease [116]. It was observed that NK/T cells could respond to glycolipids present in the context of CD1d. Because of this, anti-CD19 CAR-NK/T cells are capable of binding both CD19 and CD1d on the surface of MM cells. This results in a stronger effect than in the case of CAR T cells targeting only CD19. Additionally, CD19 CAR-NK/T cells showed low cytotoxicity against monocytes, which have the highest expression level of CD1d among blood cells. This further strengthens their therapeutic potential [116].

## 5. Limitations and Future Development Strategies for CAR T Cells in MM Treatment

After confirming the promising results of CAR T therapy for patients with MM in early-stage clinical trials, investigators have focused on developing optimal protocols. The major aims are to prevent or at least control CAR T-related side effects and toxicities and enhance the efficiency and durability of this therapy (Table 2).

The most frequently observed toxic effects of CAR T therapy are CRS, graft-versus-host disease (GvHD), and neurotoxicity (NT). The first signs of CRS occur within the first week after administration in almost 90% of patients with MM. These signs include high fever, fatigue, myalgia, nausea, and anorexia [117]. Generally, CRS is mild or moderate; however, the progression of severe CRS can lead to life-threatening complications such as severe hypotension, dysoxia, or multiple organ dysfunction [117]. CRS is induced by an abnormally high level of pro-inflammatory cytokines, such as IL-6 or IFN-γ, and granulocyte-macrophage colony-stimulating factor (GM-CSF). It was shown that CRS is associated with the expansion and activation of CAR T cells. Therefore, to relieve the toxicity of CRS, the following agents are used: IL-6 receptor antagonist (tocilizumab), GM-CSF blocker (lenzilumab), and corticosteroids [118]. Tocilizumab has demonstrated strong efficiency in treating severe CRS without negatively affecting CAR T-cell performance [119]. Another anti-CRS agent, siltuximab, prevents IL-6 from binding to its receptor by blocking it. Siltuximab is administered only when tocilizumab and corticosteroid fail to treat CRS [120].

GvHD occurs because scFvs used clinically in anti-BCMA CARs are usually murine-derived. This can induce a host immune response and, as a result, reduce both safety and efficacy. Today, many CAR-containing human scFvs has been developed to lower the immunogenicity; for example, CARs with antigen-recognition domains consisting of a fully human heavy-chain variable domain without a light-chain domain (designated FHVH33-CD8BBZ) [121].

NT resulting from CAR T-cell therapy usually occurs with new onset of neural symptoms within three weeks after CAR T-cell administration [118]. Very often, NT appears after high-grade CRS, but atypical NT events have also been observed. NT is associated with headache, apraxia, ataxia, dysgraphia, seizures, and myoclonus [119]. Unfortunately, in contrast to CRS, NT responds weakly to tocilizumab treatment [122]. Therefore, today other agents such as corticosteroids are the leading drugs for NT treatment. However, no solid data are available to support the use of corticosteroids for NT treatment [123].

Another huge concern is the adverse effects that occur from the presence of target antigen on normal cells, called the “on-target/off-tumor” effect. For instance, CD38 is present on many cells, such as plasma cells, NK cells, monocytes, T cells, and B cells, and as a result CAR T cells targeting this antigen may induce cytopenia. This indicates the strong need for CAR T cells targeting only those antigens that are present on tumor cells.

To date, various approaches have been suggested to reduce the “on-target/off-tumor” behavior of CAR T cells. One is to optimize the CAR external domain in order to lower its capability to bind to normal cells. As a result, a special class of CAR T cells can be generated, which are highly specific for tumor cells due to their high antigen density on the surface [76]. Another strategy takes advantage of the doxycycline (DOX)-regulated Tet-on system, which can simultaneously maximize “on-tumor” activity and minimize “off-tumor” effects. Such CAR T cells containing a DOX-induced structure without DOX administration are kept inactive and are activated after DOX administration [124].

Apart from side effects, another problem with CAR T-cell therapy is temporary responses and the fact that half of the patients will relapse or have disease progression after one year. Downregulation or loss of BCMA expression on the MM surface is considered as the main reason for relapse. Unfortunately, the precise mechanism for this process is still not fully understood. One possibility is the release of BCMA into the bloodstream by γ-secretase. Thus, inhibiting this enzyme may result in increased BCMA expression on MM cells and, as a result, may enhance the antitumor activity of anti-BCMA CAR T-cells. A clinical trial of anti-BCMA CAR T-cells with a γ-secretase inhibitor is ongoing (NCT03502577). Furthermore, despite that BCMA is overexpressed in MM cells, its expression level can be different among clinical samples of patients. The median BCMA expression level was 1479 specific antibody-binding capacity (SABC) units (range, 42–14,055) on malignant plasma cells and 673 SABC units (range, 189–1713) on normal plasma cells [32]. Additionally, it was shown that the loss of BCMA can be associated with CAR T-cell-induced trogocytosis, a transfer of BCMA molecules from tumor cells to the CAR T cell surface. As a result, CAR T cells start targeting each other, leading to mutual damage [125]. The problem of antigen escape is crucial, and the broader uptake of CAR T cell therapy in the treatment of MM will largely depend on how and when this problem is solved. One possible solution is to expand the palette of available antigens that can be targeted together with BCMA.

Another unmet need is to be able to improve efficacy and identify the optimal timing for the application of CAR T cells in patients. One possible solution is to use compound CAR T cells. These are T cells that express two (or more) different CARs [126]. The goal is to simultaneously target many antigens to overcome the limitation of the loss of one particular antigen [127]. For example, compound CAR T cells expressing both anti-BCMA and anti-SLAMF7 CAR have been developed, and it was shown that these cells efficiently reduced the number of BCMAs and SLAMF7-positive cells, in contrast to anti-BCMA CAR T cells alone [126].

## 6. Conclusions

CAR T-cell therapy has confirmed high potential in patients with MM. Different CAR T-cell constructs have been studied in clinical trials and have proved to have high efficacy and safety in patients with MM. However, despite the promising results of initial clinical research, a myriad of challenges have emerged. Therefore, to fully capture the potential of this therapy for MM treatment, future research should focus on the mechanisms of relapse, reduction of “off-target” toxicities, expansion of the CAR target antigen spectrum, structural improvements, and combination therapies.

## Figures and Tables

**Table 1:** Selected clinical studies of CAR T cells in multiple myeloma therapy.

Target	Phase	Study Site/Company	Status	T-Cell Dosage	No. of Patients	Efficacy	Registration Number
BCMA	1	NCI	completed	(0.3–9.0) × 10^6^ CAR+ T cells	16	ORR 81% *	NCT02215967
BCMA	1	UPenn/Novartis	completed	(1–50) × 10^7^ CAR+ T cells	25	ORR 48%	NCT02546167
BCMA	1	Celgene/Bluebird	completed	(50–800) × 10^6^ CAR+ T cells	33	ORR 85%	NCT02658929
BCMA	1	Celgene/Bluebird	ongoing	150 × 10^6^ CAR+ T cells	12	ORR 83%	NCT03274219
BCMA	1/2	Nanjing Legend	ongoing	(0.07–2.1) × 10^6^ CAR+ T cells	57	ORR 88%	NCT03090659
BCMA	1	Memorial Sloan-Kettering Cancer Center/Juno	ongoing	(72–818) × 10^6^ CAR+ T cells	11	ORR 64%	NCT03070327
BCMA	1	Nanjing Legend, China	ongoing	(0.21–1.52) × 10^6^ CAR+ T cells	17	ORR 88.2%	ChiCTR-ONH-17012285
BCMA	1	Fred Hutchinson Cancer Research Center/Juno	ongoing	(5–15) × 10^7^ CAR+ T cells	11	ORR 100%	NCT03338972
BCMA	1/2	Celgene (ex Juno)	ongoing	(50–150) × 10^6^ CAR+ T cells	44	ORR 82%	NCT03430011
BCMA	1/2	Poseida	ongoing	(48–430) × 10^6^ CAR+ T cells	19	ORR 43%	NCT03288493
K light chain	1	Baylor University	completed	(0.2–2.0) × 10^8^ CAR+ T cells	7	ORR 0%,	NCT00881920
CD138	1/2	Chinese PLA General Hospital	completed	(0.44–1.51) × 10^7^ CAR+ T cells	5	ORR 20%	NCT01886976
CD19	1	University of Pennsylvania, Novartis	completed	(1.1–6.0) × 10^8^ CAR+ T cells	10	ORR 20%	NCT02135406

BCMA, B-cell maturation antigen; CAR, chimeric antigen receptor; NCI, National Cancer Institute; ORR, overall response rate; PLA, People’s Liberation Army; UPenn, University of Pennsylvania; * 81% (13 of 16) ORR in the case of 9 × 10^6^ CAR T cells/kg (highest dose).

**Table 2:** Possible risks of CAR T cells in multiple myeloma (MM) treatment and mitigation strategies. GM-CSF, granulocyte-macrophage colony-stimulating factor; DOX, doxycycline.

Risk	Mitigation Strategy
Cytokine release syndrome (CRS)	IL-6 receptor antagonist (tocilizumab), IL-6 blocker (siltuximab), GM-CSF blocker (lenzilumab), corticosteroids
Graft-versus-host disease (GvHD)	Human scFv to reduce immunogenic potential
Neurotoxicity (NT)	Corticosteroids
“On-target/off-tumor” effect	DOX-activated CAR T cells
Lack of response to CAR T-cell therapy	Identify novel target antigens
